# Examining the Moderation Effect of Political Trust on the Linkage between Civic Morality and Support for Environmental Taxation

**DOI:** 10.3390/ijerph17124476

**Published:** 2020-06-22

**Authors:** Jae Young Lim, Kuk-Kyoung Moon

**Affiliations:** 1 Community Wellbeing Research Center, Seoul National University, Seoul 08826, Korea; jaeyounglim@yahoo.com; 2 Department of Public Administration, Inha University, Incheon 22212, Korea

**Keywords:** civic morality, political trust, environmental taxation

## Abstract

Climate change and pollution are threatening sustainable environments and human life. To mitigate and adapt to the effects of such threats, governments around the world need significant financial resources. Accordingly, this study focuses on which factors are associated with individuals’ support for taxation to protect the environment and pays special attention to the direct effects of civic morality and political trust, as well as their joint effects on support for environmental taxation. Ordered probit results with a sample size of 760 demonstrate that civic morality is positively associated with individuals’ support for environmental taxation; political trust works in the same way. More importantly, political trust moderates and enhances the linkage between civic morality and support for environmental taxation, demonstrating that it can serve as a powerful tool in a government’s efforts to protect the environment.

## 1. Introduction

The latest Intergovernmental Panel on Climate Change (IPCC) report explored the meaning behind a 1.5 °C increase in the global environmental temperature. Specifically, the experts on the IPCC warned that human activities, if continued unchanged, would result in a 1.5 °C increase between 2030 and 2052 [1]. This portends a significant number of extreme weather and associated events. Even within a 0.5 °C increase, the world has experienced an unusual number of environmental disasters [1]. Australia and the United States are already struggling with prolonged wildfires, while storms are ravaging countries from all angles and during all seasons [2,3,4]. As such, humans should adapt to and mitigate the temperature increase by changing political, social, and ecological systems, as well as making efforts to reduce greenhouse gases [5,6]. To assist with these efforts, governments need resources and taxation to support those activities, which is more urgent now than ever before. Supporting taxation for environmental protection can be categorized as pro-environmental behavior, and the factors associated with such behavior have important implications that warrant a scholarly investigation.

In recent decades, research on environmental behavior and psychology has flourished, and scholars have identified several factors as critical in explaining individuals’ pro-environmental behaviors. These include individuals’ values, norms, beliefs, attitudes, perception of threats, knowledge, locus of control (or self-control), demographic characteristics, and external factors [7,8,9,10,11,12,13,14,15,16]. Nonetheless, a few underdeveloped research areas include joint effects of multiple factors in explaining pro-environmental behaviors. Consequently, this study focuses on two of the factors and their joint effects on individuals’ support for environmental taxation: citizens’ norms related to good citizenship (i.e., civic morality) and citizens’ perceptions of their trust in politics (i.e., political trust), a major external element. As political trust plays a key role in how government policies are perceived and evaluated by citizens [17,18], it can moderate the linkage between citizens’ civic morality and their pro-environmental behaviors.

Civic morality refers to having honesty and respect toward others and the public good [19]. Individuals with a high degree of civic morality take rules, institutions, and social norms seriously and feel responsible for and obedient to them [19,20]. Because the respect is directed to those rules and norms, civic morality enables citizens to comply with them [19,20]. They do so in the public interest, even if such rules and norms are unpopular and evading them is not only legally inconsequential but also vastly advantageous [19]. Since civic morality entails civic duty and obligations, scholars have naturally found close associations between civic morality and compliance with rules and social norms, such as not evading taxes [21,22]. Others have explored the link between civic morality and pro-environmental behaviors [23,24,25,26]. Because civic morality is concerned with the public good, of which the environment is a vital part, those with a high degree of civic morality are expected to exhibit pro-environmental behaviors and support the taxation needed to protect the environment.

Political trust has relevance in contemporary society due to its decline in many countries [17]. Citizens have become increasingly cynical toward the workings of the government, and this has grave implications for how the government operates and pursues its policies. Defined as citizens’ feelings toward government relative to their expectations of how it ought to be [17,27], *political trust* serves as a heuristic, a mental shortcut for individuals who may be deficient in knowledge and have little time and few resources to understand what is happening around them [17,18,28]. For them, political trust operates as a cue by which individuals judge governments’ policies, initiatives, and legislation. Thus, high-trusting individuals are likely to benefit from doubt in the form of support of a given governmental policy without actually understanding what it does and entails. Consequently, political trust would enable high-trusting citizens to support a government action tailored to environmental protection [29,30,31,32,33], even though such action may require taxation.

More importantly, political trust in this study functions as the moderator of the linkage between individuals’ civic morality and their support for environmental taxation. Both civic morality and political trust are functional equivalents [20], and both serve the common function of engendering an obligation toward government action or policy [19,20]. Political trust as a heuristic helps individuals support government action, as they find the government trustworthy [18]. Similarly, civic morality compels individuals to follow social norms, such as paying taxes [19,20]. Thus, political trust operates as an added force that enhances potentially positive relationships between civic morality and support for taxation.

By bringing political trust to the front of explaining the relationship between civic morality and support for environmental taxation, this study combines two factors—values and perceptions induced by an external factor—in explaining pro-environmental behavior. In doing so, this study helps advance a scholarly understanding of what influences individuals’ pro-environmental behaviors.

This work will proceed as follows: First, it will introduce the theoretical mechanisms behind the direct effects of civic morality and political trust, as well as their joint effects on individuals’ support for environmental taxation. Subsequently, it develops hypotheses for an empirical analysis, followed by the presentation of the results and their implications for policymakers.

## 2. Civic Morality and Support for Environmental Taxation

Civic morality forms a foundation for a democratic society and its vitality. Also called *norms of citizenship* [20], civic morality is a sense of obligation and duty to social rules and norms [19]. This compliance is indispensable for maintaining social order; otherwise, unpopular rules and norms could only be enforced by coercive power [20]. In democratic decision-making processes, citizens’ willingness to comply voluntarily is pivotal in generating peace in everyday life [20].

In essence, civic morality implies having honesty and respect toward other citizens for the sake of the public good [19]. Because of this obligation to uphold the public interest, citizens with a greater degree of civic morality feel responsible for and compelled to follow social rules and norms [19]. Civic morality is designed to maximize public gains rather than personal gains [19]. It accepts duties owed to society and its members [19], and a keen sense of duty compels citizens to possess compliant behavior [34]. This also helps a society avoid exorbitant enforcement and deterrence costs with respect to catching and punishing law-breaking citizens [19].

Civic morality entails following a diverse set of imagined norms to be a good citizen [35,36]. These norms include regular voting, complying with the laws, being active in community affairs, and being sympathetic to the poor and unfortunate [35,36]. Moreover, rules need not be popular to be constituted as civic morality. Civic morality also applies to unpopular rules, such as paying taxes [22]. In many parts of the world, tax authorities are not equipped to detect every transaction of tax evasion. A predominant majority of citizens in developed countries pay taxes regularly, even though tax evasion seems tempting and advantageous [22]. While citizens may have an economic rationale for compliance with taxation in considering what evasion may cost them, normative considerations go beyond the economic calculus [20,37]. Citizens concerned about normative considerations prioritize ethical and moral standards that should be met [20]. By complying with a series of norms that pave the way for being good citizens, civic morality defines “an ideal citizen in a world deprived of practical limitations” [20].

Scholars have consistently found close associations between civic morality and pro-social behaviors. For instance, Torgler used the term *tax morale* to explain taxation and how it relates to tax compliance [22]. Environmental education studies have consistently linked civic education with environmental morale and activism [23]; an environmental civic duty nurtured in schools encourages individuals to form positive attitudes about sustainable development [24]. A high degree of ecological citizenship facilitates individuals’ environmentally friendly behaviors in everyday life when compared to those with a lower degree of environmental citizenship [25]. Civic cooperation is also positively associated with individuals’ willingness to pay higher taxes for environmental protection [38], as individuals possessing altruistic values influence the formation of pro-environmental attitudes [26].

These studies point to the linkage between individuals’ civic morality and their attitudes toward environmental taxation to protect the environment, making the following hypothesis plausible for an empirical examination.

**Hypothesis** **1.** *Individuals’ civic morality is associated with their level of support for environmental taxation*.

## 3. Moderating Role of Political Trust on the Linkage between Civic Morality and Support for Environmental Taxation

Political trust emerged as a vital scholarly concept in the 1970s, when countries such as the United States experienced a precipitous decline in political trust. Naturally, scholars have explored the factors leading to such decline [39,40]. Soon after, however, scholars shifted their focus to what political trust can do. As part of this, studies have begun to examine the dynamic roles of political trust as a predictor or moderator in explaining citizens’ policy preferences [17,18,27,28,41,42,43,44,45,46,47,48]. In line with this, the current study narrows its focus to the moderating effect of political trust on the linkage between individuals’ civic morality and their support for taxes to protect the environment.

Political trust can be defined in terms of three approaches: a performance-based approach, a process-based approach, and a probity-based approach. A *performance-based* definition focuses on citizens’ feelings toward government based on their expectations of what it ought to be [17]. For instance, governmental performance in handling disasters or economies can affect citizens’ confidence in political institutions. A *process-based* approach emphasizes the processes that lead to performance [49]. However, citizens may dislike conflicts and gridlocks implicated in the processes affecting government performance. A *probity-based* approach focuses on government integrity and transparency [41,45,50]. In this approach, political trust plummets amidst government scandals, but it is heightened in the absence of such scandals and corruption. Thus, political trust can be conceptualized so that it stems from people’s perceptions of government performance, processes, and integrity.

Scholars have focused on the positive roles of political trust in enhancing individuals’ perceptions of government and its policies [17,18,28]. This stems from viewing political trust as a heuristic [17,18,28]. Political trust as a heuristic serves as a simple decision rule for individuals [17,18,28]. Individuals do not possess the necessary knowledge or philosophical understanding to analyze and understand government programs nor do they often make a cognitive effort to process information sufficiently [51,52]. Thus, individuals need “cognitive shortcuts” or “rules of thumb” [18]. These helps simplify complex information and problem-solving into digestible, evaluative operations [18,53]. In the context of evaluating government policies, political trust becomes a mental cue by which individuals support or oppose government action; in so doing, individuals can then make effortless judgements [18]. With high trust levels, people will exhibit positive attitudes toward new government programs or actions; because they find the government trustworthy, they deem its actions trustworthy, and thus support them.

This heuristic function exists because people were satisfied with government performance in the past [17,18]. The heuristic is also prospective that it will facilitate individuals’ support of government action in the future [17,18]. Moreover, political trust is not always activated for individuals with respect to any government action. Rather, such action should involve some sacrifice (such as material interest) or risk from individuals [18,28,47].

Several studies have examined the relationships between political trust and individuals’ preferences for government actions or policies, and found positive associations between political trust and redistributive programs, such as welfare aids and racially targeted programs [17,28,43]. Scholars have also found similar results between political trust and individuals’ pro-environmental behaviors [29,30,31,32,33]. For instance, high-trusting individuals are likely to make sacrifices for the environment [29], as well as favor pro-environmental policies [30,31,32]. Political trust also helps individuals take ameliorative actions toward climate change [33]. These findings lend strong support to the theoretical premise that political trust functions as a heuristic for individuals, and thus would be positively associated with individuals’ support for taxation to protect the environment, particularly given that taxation involves individual risks or sacrifices activating political trust.

More importantly, this study contends that political trust can moderate and augment the relationship between individuals’ civic morality and support for environmental taxation. Political trust is a heuristic that helps individuals become willing to support government action, even if they are not fully aware of its details and consequences [17,18,28]. In essence, political trust facilitates individuals’ compliance with such action. In a similar vein, civic morality keeps a democratic society and its vitality intact by enabling citizens to comply with commonly accepted rules and norms. However unpopular, unwanted, or excruciating taxes might be, a predominant majority of citizens do not evade taxes [20,22]. Political trust and civic morality, then, serve the common goal and function of compliance with government action or policy [20]. Thus, political trust and civic morality are functional equivalents [20]. Because political trust engenders obligations toward political institutions due to heuristics giving legitimacy to the authority, it can enhance the sense of obligation individuals with a high degree of civic morality feel toward supporting environmental taxation. Conversely, if citizens are distrustful of the authority, this will erode the confidence those with a high level of civic morality will have toward taxation to protect the environment [54].

**Figure 1:**
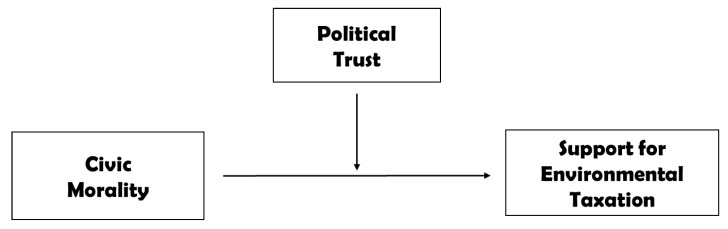
The Conceptual Framework.

Studies on political trust show that its heuristic force is potent as a moderator between ideology and policy preference, issue salience, and policy preferences, and government involvement and policy preferences [18,27,47]. Given the potency of political trust as a heuristic to help individuals support policies as well as political trust and civic morality as functional equivalents inducing citizens’ compliance with government action, political trust is likely to moderate and augment how individuals with a strong sense of civic morality perceive supporting taxation for environmental protection. These rationales form the basis for the following hypotheses for an empirical examination. Figure 1 provides the conceptual framework used in this study.

**Hypothesis** **2.** *Political trust is positively associated with individuals’ support for environmental taxation*.

**Hypothesis** **3.** *Political trust moderates and enhances the relationship between individuals’ civic morality and their support for environmental taxation so that the relationship becomes stronger when the level of political trust is higher*.

## 4. Data and Measurement

The empirical model in this study relied on the 2014 Korean General Social Survey (KGSS) [55]. The KGSS began in 2003. Its core questions and format resemble those of the U.S. General Social Survey [55]. As such, the survey targets a core group of questions repeated in every survey, complemented by topical questions repeated in every several surveys [55]. The KGSS itself has been implemented every year between 2003 and 2014, and biennially since 2014 [55]. This study used the 2014 survey because it features question items most relevant to the theory.

The response rate for the 2014 survey was 55%. The KGSS survey relies on an area probability sampling that reflects proportional population size. The sampling’s main components are the smallest administrative districts in Korea and resident registry statistics. Based on these components, the survey randomly selects individuals who are 18 years or older. The survey also excludes individuals who are institutionalized [55].

In terms of respondents’ demographics, 42% of respondents were female, while 58% were male. The average of respondent age was approximately 45 years-old. The average income level of respondents was 6.46, earning before-tax income ranging between KRW 2,500,000 and KRW 2,990,000. The average educational level of respondents was 4 (technical college education that provides 2 years of higher education).

### 4.1. Dependent Variable

The dependent variable in this study is support for environmental taxation. The variable relies on a single, subjective item: “How willing would you be to pay much higher taxes to protect the environment?” [55]. Scoring for the item ranges from 1 to 5. Signaling strong support, 5 indicates a “great willingness”, while 1 indicates a “great unwillingness,” signaling strong opposition. The mean was 3.33, slightly above 3, showing that, in general, respondents possessed slightly more favorable attitudes toward paying taxes for environmental protection.

While a single item is not ideal due to a lack of psychometric properties that multi-item measures can afford, several studies have demonstrated that a single-item measure can approximate a multi-item measure due to high correlations between the two [56,57]. Similarly, a subjective item denoting behavioral intentions is not equal to an objective item denoting actual behaviors, but some studies have displayed high correlations between the two measures [7,58], suggesting that a subjective item can be an alternative to an objective item when objective measures are not readily available.

### 4.2. Explanatory Variable

#### 4.2.1. Civic Morality

Civic morality consists of six items, the scoring for each of which ranges from 1 to 7. Respondents were asked to reveal how important each of the following six items is to being a good citizen: voting in every election, no tax evasion, monitoring government actions, understanding others with different views, choosing products ethically, and helping other worse-off Koreans. The measure was reliable, with Cronbach’s α equaling 0.768. The mean of the variable was 5.57, suggesting that respondents generally possessed a relatively higher degree of citizenship norms. As hypothesized, civic morality was expected to be positively associated with individuals’ support for environmental taxation.

#### 4.2.2. Political Trust

Political trust comprises four items, the scoring for each of which ranges from 1 to 3. Respondents were asked to reveal their confidence in the following four institutions: central agencies, the National Assembly of Korea, the president’s office (Cheongwadae), and the local government. The measure was highly reliable (Cronbach’s α= 0.809). The mean of the variable was 1.45, closer to the minimum level of trust than the maximum, suggesting that respondents generally did not possess a high degree of confidence in the country’s four major political institutions. As hypothesized, political trust is expected to be positively associated with individuals’ support for environmental taxation and to moderate and enhance the relationship between individuals’ civic morality and their support for environmental taxation.

### 4.3. Controls

The model also accounts for several control variables. First, the model included political participation. Political participation touches on individuals’ self-assessment of their political life. The measure consists of four items. Respondents were asked to assess their participation in (1) political rallies, (2) demonstrations, (3) product boycotts, and (4) online forums. The measure was highly reliable (Cronbach’s α= 0.951). Several studies have found that political participation generates a strong sense of locus of control and self-efficacy, with which individuals are likely motivated to pursue desirable individual, social, and political outcomes [59,60]. Thus, individuals with a greater level of political participation are more likely to pursue ameliorative actions with respect to the environment [13,61,62]. Second, the model accounts for individuals’ political interests. The measure relies on a single item; respondents were asked how interested they personally are in politics. Individuals with a strong interest in politics are more likely to gain in-depth knowledge on contemporary problems, such as the degree to which pollution and climate change threaten the environment, and to support an action to mitigate them [63]. In this sense, they would likely be well-disposed to support taxation to protect the environment. Third, the model accounts for a perceptual measure of environmental threat. The measure comprises six items: respondents’ perceptions of vehicle-induced air pollution, industry-induced air pollution, farming-induced pollution, water pollution, global warming, and gene-modified crops. The measure was highly reliable (Cronbach’s α= 0.984). Several studies have demonstrated that individuals exhibiting a high degree of threat perception display positive orientations toward resolving such threats [64,65]. Since environmental protection is gaining urgency in South Korea due to frequent occurrences of fine dust and temperature warming [66], those feeling threatened by such events would be supportive of ameliorative actions, such as taxation, to protect the environment. Fourth, the model includes additional perceptual measures about how individuals perceive environmental threats in their community. The measure consists of three items: Respondents were asked how they perceive (1) air, (2) water, and (3) noise pollution in their communities. There exist strong connections between individuals’ direct experiences with the environment near their homes and their support for a remedial action [12]. This prompts the expectation that individuals perceiving a greater degree of threat near where they live are more likely to be motivated to support taxation that addresses environmental protection. The measure was also highly reliable (Cronbach’s α= 0.989).

Finally, the model accounts for several demographic variables. Studies have shown that women tend to support environmental causes and display pro-environmental behaviors more than men [67]; as people grow older, they find themselves closer to the environment [68]; the well-educated possess necessary knowledge and understanding with which they display positive attitudes toward efforts to protect the environment [69]; and high-income earners are likely to be equipped with sufficient resources and time to invest more in environmental causes than low-income earners [30]. Income and education are expressed as levels. Respondents were asked to choose a level to which they belonged for their education and income (before-tax average monthly income). In terms of education, 0 indicates “no formal schooling”; 1, “elementary school”; 2, “junior high school”; 3, “high school”; 4, “junior college” (two-year course); 5, “college” (four-year course); 6, “graduate school (master’s degree)”; 7, “graduate school (doctoral degree).” In terms of monthly income, 0 indicates “no income”; 1, “less than KRW 500,000”; 2, “KRW 500,000-990,000”; 3, “KRW 1,000,000–1,490,000”; 4, “KRW 1,500,000–1,990,000”; 5, “KRW 2,000,000–2,490,000”; 6, “KRW 2,500,000–2,990,000”; 7, “KRW 3,000,000–3,490,000”; 8, “KRW 3,500,000–3,990,000”; 9, “KRW 4,000,000–4,490,000”; 10, “KRW 4,500,000–4,990,000”; 11, “KRW 5,000,000–5,490,000”; 12, “KRW 5,500,000–5,990,000”; 13, “KRW 6,000,000–6,490,000”; 14, “KRW 6,500,000–6,990,000”; 15, “KRW 7,000,000–7,490,000”; 16, “KRW 7,500,000–7,990,000”; 17, “KRW 8,000,000–8,490,000”; 18, “KRW 8,500,000–8,990,000”; 19, “KRW 9,000,000–9,490,000”; 20, “KRW 9,500,000–9,990,000”; and 21, “more than KRW 10,000,000.” Table 1 displays the variables’ descriptive statistics.

### 4.4. Measurement Validity

Because the study relies on a single-source dataset, it is susceptible to common method variance. Thus, we have employed a series of checks to ensure that the model does not suffer from common source bias. First, Harman’s single-factor test was implemented to find whether a single factor dominated respondents’ answers to other factors included in the model. The test only found 17.33% of one factor in explaining the covariance in the model. Second, confirmatory factor analysis (CFA) was performed to identify a model fit. Included in the analysis were five factors: civic morality, political trust, political participation, perceived local pollution, and perceived environmental threats. The analysis produced the following fit indices: $\chi^{2}=$ 876.985, RMSEA = 0.047, SRMR = 0.034, CFI = 0.939, and TLI = 0.930. The numbers meet generally accepted threshold values [70], suggesting that the five-factor model fit the data well.

## 5. Results

The empirical analysis employed an ordered probit. Because the dependent variable contains ordinal values, running a linear regression can produce biased estimates. A binary probit is an alternative method, but it shrinks information contained in the ordinal values. Thus, an ordered probit was used for the empirical analysis. Finally, the analysis accounted for robust standard errors, as well as a weight that represented the population.

The results are displayed in Table 2. The empirical analysis proceeded in two steps. In the first step, only direct relationships between the explanatory variables and dependent variable, as well as between the controls and dependent variable, were considered. The second step was centered on identifying the joint effects of civic morality and political trust on individuals’ support for environmental taxation.

The results confirmed the three hypotheses. First, individuals’ civic morality was positively associated with their support for environmental taxation. Those who hold citizenship norms in high esteem are likely to exhibit compliance with taxation and demonstrate positive attitudes toward the environment. Second, high-trusting individuals were also positively associated with their support for environmental taxation. High-trusting individuals are more likely to view governmental causes in a favorable light and be supportive of taxation that funds such causes. More importantly, the study focused on whether political trust moderates and augments the linkage between individuals’ perceptions of civic morality and their support for environmental taxation. The results showed that political trust enhanced and amplified the linkage. Because political trust functions as a heuristic to compel individuals to support environmental taxation, it enhances the sense of obligation to comply with environmental taxation for those possessing a higher level of civic morality.

In terms of the controls, several variables behaved as previous findings have suggested. Individuals’ degree of political participation and interest was positively associated with supporting environmental taxation [59,60,63]. Individuals with a heightened perception of environmental threat were also positively associated with support for environmental taxation [64,65]. Similarly, individuals showed more favorable attitudes toward supporting environmental taxation when they possessed a greater perception of local pollution near their places of residence [12]. Two demographic variables—income and education—were also positively associated with supporting taxes for environmental protection [30,69].

Table 3 displays the predicted probabilities of individuals strongly supporting environmental taxation (the value of the dependent variable = 5). This demonstrates the difference with respect to “strong support” between when a variable is set at the minimum and when it is set at the maximum. For the interaction, the difference is between when both variables are at their minimum and when they are at their maximum. The heightened sense of civic morality shifted “strong support” for environmental protection by 10.79%. The influence of political trust was less substantial than that of civic morality, yet it was more than 7%. The focus of the study on the moderation effects of political trust, however, was justified when examining the joint effects of civic morality and political trust on “strong support” for environmental taxation. The shift yielded more than a 34% change for “strong support.” Thus, both variables became more powerful in their combination with “strong support.”

**Figure 2:**
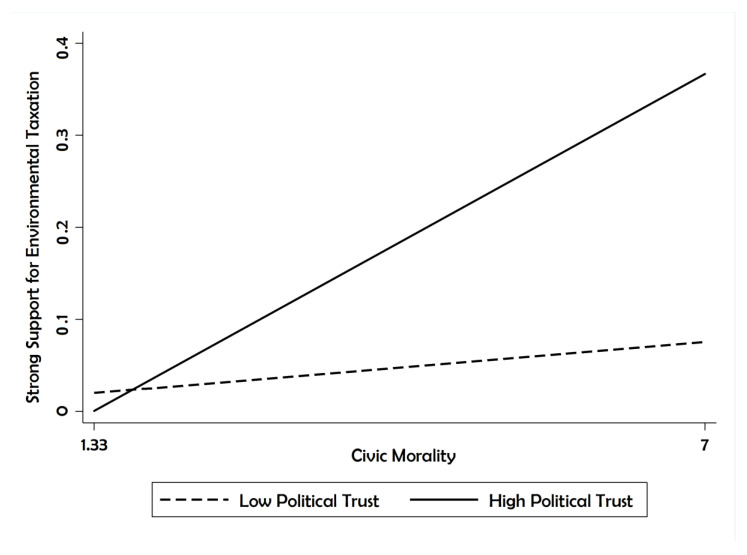
Predicted probabilities of strong support for environmental taxation.

Figure 2 visualizes the joint effects of civic morality and political trust on having strong support for environmental taxation. The solid line refers to the relationship between civic morality and “strong support” for environmental taxation when the level of political trust is at its maximum (high trust). The dashed line denotes the linkage when the level of political trust is at its minimum (low trust). The slope becomes much steeper when the level of political trust is high, whereas the slope changes little when the level of political trust is low. Once again, the joint effects of civic morality and political trust are noticeable.

## 6. Discussion

The study focused on the direct effects of civic morality and political trust as well as their joint effects on individuals’ support of environmental taxation. The results showed that civic morality and political trust were positively associated with support for environmental taxation, and more importantly, they jointly exerted a powerful influence on support for environmental taxation. The results help expand on the existing body of environmental behavior research. Studies on individuals’ pro-environmental behaviors have explored a wide array of factors. For instance, they have identified values and beliefs, perceptions, loci of control (internal vs. external), attitudes, knowledge, demographic characteristics, and external circumstances [7,8,9,10,11,12,13,14,15,16]. While these have helped illuminate why individuals behave in a positive manner toward the environment, few have examined the role politics plays in terms of how many individuals place their trust in major political institutions. If civic morality can be characterized as valuing others, political trust can be considered values or perceptions induced by an external circumstance known as politics. The study brings together two elements that make up the factors associated with the determinants of pro-environmental behaviors. Examining political trust is also relevant in that individuals’ behavior is significantly dampened or stimulated by their perceptions of politics. When ideological battles or gridlocks seem to intensify across countries, political trust has significant implications for how citizens approach major issues, such as climate change and pollution, which are vital for a sustainable environment and human life.

The results suggest important implications for policymakers. First, the results proved that civic morality is a critical element in citizens’ support for pro-environmental behaviors. Thus, it is important for policymakers to direct their attention to improving citizens’ sense of civic morality. Scholars have been concerned about nurturing civic duty among citizens for quite some time [23,25,26]. One of the ways they have argued to boost citizens’ civic morality begins with developing their political self-efficacy. This is based on the premise that citizens isolated from political life develop skeptical worldviews and negatively view values and principles that can hold society together [29,50]. Improving citizens’ self-efficacy can start in the school setting [12,13]; students need to learn about facilitating their self-efficacy. They can be nudged toward this through various mechanisms, including open-ended discussions to learn how to freely express themselves, working with other politically oriented groups to feel a sense of bonding for a common purpose, and participating in simulated activities, such as mock elections and demonstrations [13]. Studies have repeatedly shown that individuals with a stronger sense of self-efficacy develop internal efficacy (i.e., the belief that they can affect personal and political changes) and external efficacy (i.e., the belief that they can demand change from the government) to bring about positive changes to themselves and their society [59,60]. Pro-environmental policies and behaviors are an urgent need in this age of global warming, and they can emerge from people’s moral principles about being an active citizen.

Second, the results suggested that political trust plays a crucial role in facilitating citizens’ support for environmental taxation and augmenting the linkage between citizens’ civic morality and their support for environmental taxation. Increasing citizens’ confidence in their country’s political institutions can bring positive effects to environmental protection efforts. Scholars have extensively studied the factors associated with political trust [17,28,39,40], identifying factors such as government corruption, perception of the legislative body, and performance-based explanations [17,28,39,40,42]. For instance, government corruption dampens public confidence in mainstream politics. In South Korea, the corruption-ridden Park administration led to massive candlelight demonstrations for months and the president’s eventual ousting in 2017 [71,72]. Government performance is also a crucial element in explaining political trust. Citizens’ perceptions of economic performance are identified positively with political trust [17]. Economic performance is also asymmetrical, in that its salience is more pronounced in a sour economy than in a healthy economy [17]. This explains why a sour economy frequently leads to the defeat of incumbent administrations, whereas a healthy economy does not necessarily correspond to the public holding incumbent administrations in high esteem [17]. U.S.-based studies have also revealed that relative to perceptions of a president and central government, perceptions of a legislative body explain political trust to a higher degree. Parliamentary activities are exposed to the public every day, making a legislative body vulnerable to public anger [17,28]. Thus, it is important for political trust levels to be high, so all major political institutions work together, maintain integrity, and serve the public good. Indeed, this is a tall order.

A major problem lies in how to improve citizens’ political trust when it is at its all-time low in countries such as the United States [73]. It is difficult to foresee a reversing trend anytime soon; however, even with a general declining trend, a glimpse of hope remains in relation to frequent fluctuations in political trust sparked by major natural or human-caused events. For instance, the degree of political trust in the central government in South Korea was plummeting, an ominous sign for the upcoming parliamentary election, due to slow economic growth and scandals involving a presidential appointee [74]. Then, coronavirus emerged, and thousands of COVID-19 cases further dampened citizens’ confidence in the central government [75]. Within a few months, however, thousands of cases have turned into dozens, mostly induced by inbound visitors, producing a remarkable turnaround in political trust just in time for the government party to win a landslide victory, the largest such gain in modern Korean history [76]. How to maintain and improve citizens’ political trust is up to the government in coming months, but the Korean episode points to the fact that many events can influence citizens’ political trust. Citizens’ political trust levels are not fixed, but malleable by unforeseen events, and political leaders should be motivated to pursue their favorite policies when such opportunity strikes.

## 7. Conclusions

Global warming signals difficulties for human life in the years ahead [1,5]. Countries around the world have observed frequent environmental degradation and devastation, with storms, heat waves, and wildfires filling newspaper pages and TV programs [2,3,4]. An IPCC report pictured what the world will look like with a 1.5 °C increase [1]. Some of its effects will include a decline in the number of communities relying on small islands, coasts, and farming [1]. Developing countries—compared to developed ones—are especially vulnerable to the effects of global warming [1]. Some in hot-weather regions will undergo slow economic growth and a shortage of water supply [1]. All of these demand urgent responses to mitigate and adapt to global warming, with government resources directed toward the environment.

Finally, it should be noted that the study has its limitations. First, the study relied on a single-year cross-sectional dataset. Thus, the causal chain between the explanatory and dependent variables is much weaker than when using panel or experimental data. This may be inherent in cross-sectional surveys such as the KGSS, in which the same individuals are not tracked across years. Second, the dataset was collected during the same time period. Because there was no time difference between the explanatory and dependent variables, the study could not resolve any potential endogeneity between them. Third, the study relied on a single-source dataset. While a few cautionary tests (Harman’s single-factor and CFA tests) were undertaken to mitigate concerns about common method variance, the threat of common method bias may always be present when using a single-source dataset. Fourth, the study examined a single-country survey, and its applicability may be limited when compared to what a comparative survey covering several countries can offer. Finally, the dependent variable denoted behavioral intentions. While some claim close correlations between behavioral intentions and actual behavior [7], the utility of the results would be further preserved if actual behaviors were observed and recorded.

## Figures and Tables

**Table 1:** Descriptive statistics.

Variables	N	Mean	S.D.	Min.	Max.
Support for environmental taxation	760	3.33	1.10	1	5
Civic morality	760	5.57	1.01	1.33	7
Political trust	760	1.45	0.46	1	3
Political participation	760	1.67	0.66	1	4
Political interest	760	2.44	0.78	1	4
Perceived local pollution	760	2.45	0.63	1	4
Perceived environmental threats	760	3.62	0.63	1.67	5
Age	760	44.98	13.19	18	83
Female	760	0.42	0.49	0	1
Income	760	6.46	4.24	0	21
Education	760	4.00	1.39	0	7

**Table 2:** Regression results.

Variables	Support for Environmental Taxation			
	Step 1		Step 2	
	Coef.	(S.E.)	Coef.	(S.E.)
Civic morality	0.19	0.05 ***	−0.11	0.13
Political trust	0.24	0.09 ***	−0.98	0.48 **
Civic morality × political Trust			0.22	0.08 ***
Political participation	0.25	0.07 ***	0.27	0.07 ***
Political interest	0.14	0.06 **	0.12	0.06 *
Perceived local pollution	0.13	0.07 *	0.13	0.07 *
Perceived environmental threats	0.16	0.08 **	0.15	0.08 *
Age	0.00	0.00	0.00	0.00
Female	−0.04	0.09	−0.03	0.09
Income	0.02	0.01 **	0.02	0.01 **
Education	0.08	0.03 **	0.08	0.03 **
τ_1_	2.08	0.45	0.35	0.82
τ_2_	2.98	0.45	1.25	0.82
τ_3_	3.47	0.46	1.75	0.83
τ_4_	5.17	0.47	3.46	0.83
Log likelihood	−985.82		−982.53	
Wald test	117.68		130.83	
Number of cases	760			

Note: * *p* < 0.1, ** *p* < 0.05, *** *p* < 0.01.

**Table 3:** Predicted probabilities of strong support for environmental taxation.

Variables	Strong Support for Environmental Taxation		
	Minimum	Maximum	Difference
Civic morality × political trust	1.98%	36.68%	34.70%
Civic morality	0.89%	11.68%	10.79%
Political trust	5.55%	13.21%	7.66%
